# Awareness of Poultry Farmers of Interconnected Health Risks: A Cross-Sectional Study on Mycotoxins, Biosecurity, and Salmonellosis in Jimma, Ethiopia

**DOI:** 10.3390/ani14233441

**Published:** 2024-11-28

**Authors:** Tadele Kabeta, Tadele Tolosa, Alamayo Nagara, Ilias Chantziaras, Siska Croubels, Filip Van Immerseel, Gunther Antonissen

**Affiliations:** 1 School of Veterinary Medicine, College of Agriculture and Veterinary Medicine, Jimma University, Jimma P.O. Box 307, Ethiopia; tadeletolosa@yahoo.com (T.T.); dhunfanagara@gmail.com (A.N.); 2 Faculty of Veterinary Medicine, Department of Pathobiology, Pharmacology and Zoological Medicine, Ghent University, 9820 Merelbeke, Belgium; siska.croubels@ugent.be (S.C.); filip.vanimmerseel@ugent.be (F.V.I.); 3 Faculty of Veterinary Medicine, Veterinary Epidemiology Unit, Ghent University, Salisburylaan 133, 9820 Merelbeke, Belgium; ilias.chantziaras@ugent.be; 4 Faculty of Veterinary Medicine, Ghent University, Chair Poultry Health Sciences, Salisburylaan 133, 9820 Merelbeke, Belgium

**Keywords:** awareness, biosecurity, Ethiopia, Jimma, mycotoxin, poultry, Salmonella

## Abstract

Poultry farming in Ethiopia is crucial for food security and income, but it faces significant challenges due to gaps in farmer awareness. A cross-sectional study was conducted using the Biocheck.UGent™ biosecurity scoring system and a questionnaire to evaluate poultry farmers’ basic and practical knowledge concerning salmonellosis and mycotoxins. The questionnaire revealed substantial gaps in basic and practical knowledge regarding *Salmonella* spp infections and mycotoxin among 38 poultry farmers in Jimma. About 68.4% of farmers were unaware of the impact of salmonellosis on both poultry and human health. Moreover, 78.9% had limited basic knowledge of how salmonellosis affects production and the economy. Farmers also showed limited practical knowledge of farm management and hygiene practices essential for preventing *Salmonella* spp. infections. Regarding mycotoxins, 63.2% of farmers lacked awareness of poultry feed management, 60.5% were unaware of the health risks mycotoxins pose, and 73.7% did not recognize signs of mycotoxin contamination. Although 55.3% of farmers demonstrated acceptable practical knowledge of strategies to reduce the impact of mycotoxin contaminations, there are still concerns, particularly since 65.8% and 55.3% only showed moderate practical knowledge of feed storage and mycotoxin prevention, respectively. The overall biosecurity scores of poultry farms were below the global average, with a score of 41.7 compared to the worldwide average of 64. The overall mean score for external biosecurity was 44.9, below the global average of 63. All 3 scoring platforms and biosecurity parameters indicated that internal biosecurity was the weakest aspect, with a score of 31.6, well below the global standard of 64. The results showed a weak correlation (rₛ = 0.06) between farmers’ basic and practical knowledge scores about *Salmonella* spp. infections and mycotoxins. Similarly, there was a weak correlation between the poor biosecurity score of poultry farms and the basic and practical knowledge of poultry farmers on *Salmonella* spp. infections (rₛ = 0.17) and mycotoxins (rₛ = 0.25). In conclusion, the study found that poultry farmers in Jimma had poor basic and practical knowledge scores on *Salmonella*, mycotoxins, and biosecurity measures. Thus, awareness creation is paramount to improve these gaps to reduce the impact of *mycotoxin* contamination and poultry diseases and consequently to improve food security and food safety.

## Simple Summary

The assessment of farmers’ basic and practical knowledge regarding major health risks, such as poultry salmonellosis and mycotoxins, alongside the biosecurity status of farms, is crucial for effectively preventing and controlling poultry diseases in Ethiopia, where poultry production plays a vital role in community livelihoods. This research, conducted from March 2022 to June 2023, aimed to evaluate poultry farmers’ basic and practical knowledge concerning salmonellosis and mycotoxins and assess the biosecurity status of small- and medium-scale poultry farms in Jimma, Ethiopia. The study revealed a low overall level of basic knowledge and poor practical knowledge among poultry farmers of managing and handling feed to reduce mycotoxin contamination and prevent *Salmonella* spp. infections. Furthermore, biosecurity scores were significantly below global averages, with most external biosecurity parameters and all internal measures failing to meet international standards. The findings highlighted weak biosecurity measures, inadequate awareness, and suboptimal practices among poultry farmers in Jimma. Identifying these gaps in basic and practical knowledge as well as biosecurity measures will be critical in designing and implementing effective mitigation strategies for infectious diseases in poultry farms. These results underscore the need for active engagement from all stakeholders in poultry production to boost the sector’s productivity in Ethiopia.

## 1. Introduction

In Ethiopia, poultry production plays a crucial role in animal agriculture, driven by the growing demand for poultry meat and eggs, which are important sources of protein and income [1,2]. Poultry farming significantly contributes to food security and economic stability [3,4]. However, the poultry industry in Ethiopia is confronted with a complex web of challenges that significantly hinder its productivity and sustainability [4,5]. Chief among these are the widespread issue of *Salmonella* spp. infections, mycotoxins contamination of poultry feed, and inadequate biosecurity measures [6,7,8,9].

Poultry salmonellosis, caused by various serotypes of *Salmonella*, poses substantial challenges to Ethiopian poultry farms, including increased mortality, reduced productivity, higher antimicrobial usage and serious public health concerns due to the pathogen’s zoonotic nature [5,10,11,12]. A meta-analysis conducted by Kabeta et al. [13] reported a pooled prevalence of 15.7% for poultry salmonellosis in Ethiopia, highlighting its significant impact on both public health and the poultry industry. Infection rates varied widely, ranging from 2.65% as reported by Tadesse et al. [12] to 59.68% by Kebede and Duga [8] in the country. Additionally, *Salmonella* spp. accounted for 5.7% of the overall pooled prevalence estimates of foodborne diseases in Ethiopia [14]. 

Among the key factors contributing to the spread of infectious diseases like salmonellosis is mycotoxin contamination, resulting from mold growth in raw materials and poultry feed. Mycotoxins primarily compromise poultry health by weakening the immune system, making birds more susceptible to infections [15,16,17]. Inadequate biosecurity practices further exacerbate the spread of *Salmonella* spp. both within and between farms [9]. These interconnected challenges create a vicious cycle, where poor feed quality, lack of biosecurity and disease prevalence mutually reinforce each other, ultimately jeopardizing food security and safety.

Mycotoxigenic fungi and their associated toxins pose significant global concerns, with substantial economic and health impacts, especially in developing countries like Ethiopia [18]. Mycotoxin contamination compromises feed quality and reduces feed efficiency. Birds consuming contaminated feed exhibit poorer growth rates and feed conversion ratios and exhibit reduced egg production [17,19,20]. Exposure to mycotoxins also increases the susceptibility of poultry to various diseases, impairing the birds’ immune system, rendering them more vulnerable and reducing their overall resilience [15,21]. Antonissen et al. [22] observed that feeding a deoxynivalenol contaminated diet (3.5 mg/kg feed) may modulate the spread of a *Salmonella* infection in a pigeon flock by increasing the number of pigeons shedding the bacterium. Recently Liu et al. [23] demonstrated that a short-term exposure to subclinical levels of combined fumonisins (14 mg/kg feed), deoxynivalenol (0.6 mg/kg feed), T-2 toxin (0.6 mg/kg feed) and neosolaniol (0.8 mg/kg feed) had a negative impact on the intestinal tight junction proteins and increased the cecal *Salmonella* load with 1.5 logs compared to the negative control in broiler chickens. 

Mycotoxin contamination in human food and animal feed is prevalent across African countries primarily due to defects in storage and post-harvest handling practices [24]. The warm and humid conditions typical of sub-Saharan Africa heighten the risk of mycotoxins contamination, as these environments are conducive to fungal growth [25,26]. Despite the serious threat to human and animal health, public awareness and understanding of the risks and the importance of prevention remain minimal, even in the most affected areas of East Africa. Moreover, many stakeholders in the region’s food production chain are unaware of the health and economic consequences of consuming contaminated food [27]. Managing mycotoxins is crucial due to environmental factors, infrastructural deficits, informal market structures, and improper cultural practices that increase exposure risks [26,28]. The contamination of feed leads to significant waste, as highly contaminated feed must be discarded, resulting in resource loss and higher costs for poultry producers, while malpractices among feed millers further exacerbate the socio-economic impact [29].

Another critical challenge is the lack of adequate biosecurity measures. Effective biosecurity is essential for preventing the introduction and spread of infectious diseases such as salmonellosis within and between poultry farms thus safeguarding the health of the flock and minimizing the economic impact on stakeholders [30,31]. It protects against direct losses such as mortality and decreased productivity and helps mitigate indirect economic effects such as increased veterinary costs and reduced marketability of poultry products [32]. Biosecurity is vital for improving chicken production and ensuring the quality of poultry products by controlling farm access and maintaining sanitation [33,34,35]. It encompasses measures such as isolation for suspected animals to avoid or minimize contamination, cleaning to remove organic matter, and disinfection to eliminate pathogens. Strict access control is also a key component of an effective biosecurity strategy [36].

In Ethiopia, poor awareness and practices among all stakeholders, including farmers, traders, food producers, and decision-makers, contribute to widespread mycotoxin contamination in animal feed [37,38]. Similarly, biosecurity practices in Ethiopian poultry farms are inadequate, with limited implementation and poor overall biosecurity scores [39,40]. Even when awareness exists, it seldom translates into effective action due to insufficient mechanisms for monitoring and controlling mycotoxins and weak biosecurity implementation [38,41]. These gaps are compounded by high disease prevalence, including frequent salmonellosis outbreaks [42,43]. The lack of stringent biosecurity measures, coupled with poor hygiene, substandard housing, and inadequate management, along with inadequate monitoring, testing and vaccination for *Salmonella* spp. further perpetuates these challenges. Improving biosecurity awareness, practices, infrastructure, and veterinary access is crucial for enhancing the health and performance of Ethiopia’s poultry industry [5,32].

The challenges related to poor feed quality, inadequate biosecurity, and prevalent diseases hamper poultry farming productivity and sustainability in Ethiopia. Understanding the factors contributing to these issues is essential for mitigating disease occurrence. This paper aims to assess Ethiopian poultry farmers’ awareness of poultry salmonellosis, mycotoxins and biosecurity practices to explore the connections between mycotoxin contamination, biosecurity lapses and *Salmonella* spp. infections, and discuss their impact on poultry production. The study highlights the need for integrated approaches to improve health and productivity by identifying current basic knowledge and practical knowledge gaps. Farmers’ understanding of mycotoxins, biosecurity, and proper farm practices is vital in preventing contaminated feed and *Salmonella spp*. outbreaks. 

## 2. Materials and Methods

### 2.1. Study Areas, Period of Study, and Populations

This study was conducted between March 2022 and June 2023 on small- and medium-scale (50–3500 chickens) poultry farms in Jimma, Ethiopia. Jimma is situated 352 km from the capital city, Addis Ababa. The town experiences a “Woina-Dega” climatic condition, with an average altitude of 1780 m above sea level, and receives annual rainfall that ranges between 1138 and 1690 mm. The annual mean temperature varies from approximately 14 °C to 30 °C. In 2021, there were 45 poultry farms registered in Jimma; however, only 38 of these farms were operational. According to the Jimma Town Livestock Office (2021), the remaining 12 farms have closed due to challenges, such as rising feed prices, financial constraints, and disease-related issues.

### 2.2. Study Design

A cross-sectional study design and census sampling method were utilized for all available poultry farms due to the small number of farms actively involved in poultry production in the area. All active farms participated voluntarily in the questionnaire survey.

### 2.3. Data Collection

In this study, demographic information was collected, covering the farmers’ address, age, sex, occupation, education level, and experience in poultry management. The questionnaire comprised 50 yes-or-no questions, with 30 assessing basic knowledge and 20 evaluating practical knowledge related to mycotoxins. Additionally, there were 15 questions assessing basic knowledge and 15 questions on practical knowledge concerning poultry salmonellosis. For ease of understanding, the questionnaire was translated into the local language and pretested on two farms. Biosecurity scores were determined using the Biocheck.UGent™ tool, a scientific, risk-based, and independent tool for evaluating biosecurity practices on farms. The Biocheck.UGent™ online survey score platform was utilized to assess biosecurity on 38 farms. Specifically, the backyard scoring platform was used on 21 farms, while the broilers and laying hens platforms were used on 4 and 13 poultry farms, respectively. Interviews were conducted by the principal investigator with a contact person, usually, the farm owner or manager, and all 38 farmers who consented to participate were interviewed.

### 2.4. Operational Definition

Semi-intensive systems: These are a combination of extensive and intensive systems in which birds are confined to a certain area and have access to shelter.

Small scale is defined as poultry keeping by households using family labor and, wherever possible, locally available feed resources, typically to maintain a flock of fewer than 100 birds. 

Medium-scale poultry farming involves commercial production aimed at supplying both local and broader markets, often with better integration into formal market structures, and typically involves flocks of fewer than 5000 birds [44,45]. 

In this study, the term “backyard platform” was specifically used in biosecurity scores for semi-intensive farms. The online biosecurity assessment using the Biocheck scoring system allows for the evaluation of semi-intensive farms operating with a backyard platform.

Basic Knowledge: Encompasses questionnaire items used to assess the awareness and understanding of poultry farmers regarding mycotoxins and salmonellosis.

Practical Knowledge: Questionnaire items used to assess the hands-on skills and practices that poultry farmers use to prevent mycotoxin contamination and salmonellosis occurrence and manage their impact in their flocks.

Woina-Dega Zone: refers to areas with altitudes between 1500 and 2300 m.

### 2.5. Data Analysis

The data generated from the study were arranged, coded, and entered into a Microsoft Excel spreadsheet (Microsoft Office Excel 2019) before being exported to SPSS version 28, IBM Corp, Armonk, NY, USA [46]. The analysis focused on the correlation between poultry farmers’ basic and practical knowledge scores on mycotoxins and poultry salmonellosis. Basic and practical knowledge variables assumed to have a similar influence on the potential risk on the farm were combined into a single variable. Then, the yes/no responses were converted into numerical scores, where 1 point was assigned for correct answers, and 0 points were assigned for incorrect responses. The scores for each respondent were summed to create composite scores for knowledge and practice. These composite scores were divided into 3 categories within a 6-point scale: lower/poor (0–2 points), medium/moderate (3–4 points), and higher/good (5–6 points). The scores were then categorized as below-average or above-average (mean) to assess the correlation between the three issues. Respondents scoring below the mean were coded as 0, indicating “poor/unacceptable,” while those scoring above the mean were coded as 1, indicating “good/acceptable” [47]. The biosecurity scores of the farms were provided automatically after filling in the questions on the website of Biocheck.Ugent™ (https://biocheckgent.com/en, Accessed on 4 June 2023) by a scientific risk-based scoring system (Ghent University, Merelbeke, Belgium). Farms scoring below the world standard (WS) were classified as having below WS (poor) biosecurity, while those exceeding the standard were classified as having above-minimum WS (good) biosecurity scores. The Spearman correlation coefficient (rₛ) between basic and practical knowledge on salmonellosis, mycotoxins, and biosecurity were categorized as follows: weak (0–0.25); fair (0.25–0.5), good (0.5–0.75), and perfect (0.75–1) [48]. Descriptive analysis was used to describe the results in terms of frequency and proportion for all scores. Mean and standard deviation (SD) were also used to describe the results. *p*-values with a 95% CI and 5% precision were used to measure the association between determinant and independent factors.

## 3. Results

### 3.1. Socio-Demography

**Figure 1:**
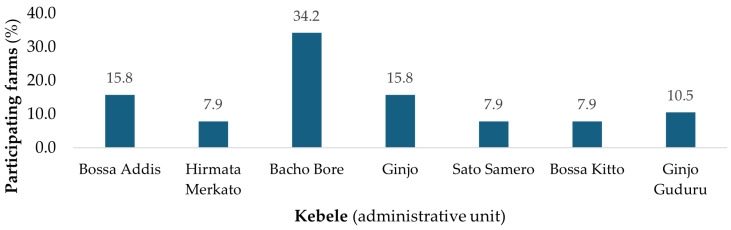
Distribution of poultry farms by kebele in Jimma.

Among 38 poultry farmers, 13 (34.2%) were found in Bacho Bore, a small administrative unit (Kebele) of Jimma (Figure 1). The majority of the respondents were female (52.6%). The mean age of the study participants was 41.5 ± 10.4 (mean ± SD) years. Among the respondents, 21% had an educational level from grades 5–8 (primary school), 42% had completed grades 9–12 (secondary school), and 37% were college/higher education graduates. Among the respondents, 39% were working as farm managers, while the remaining 61% were in other professional roles, with most of the farmers having an average of 5 years of work experience.

### 3.2. Basic and Practical Knowledge of Poultry Farmers About Salmonellosis and Mycotoxins

The survey results revealed that 68.4% of the farmers had poor basic knowledge about the impact of salmonellosis on chickens, including food-borne illnesses related to *Salmonella*, the risk posed by chicken manure in contaminating farms with *Salmonella* spp. and the effectiveness of good biosecurity practices in preventing its infections. Additionally, 78.9% of respondents had a poor understanding of the impact of salmonellosis on poultry farm production. They did not recognize that *Salmonella* spp. infections lead to economic losses due to increased mortality and morbidity, reduced weight gain, lower feed intake, decreased productivity and growth rates, and a decline in egg production (Table 1).

Regarding practical knowledge, 68.4% of farmers had poor practical knowledge of farm management and hygiene practices to prevent salmonellosis. This practical knowledge included regularly cleaning poultry houses, placing foot baths at farm entrances, using chemical disinfectants, controlling people’s movement to avoid contamination, and taking action to control rodents and pests on the farm. Moreover, 50.0% of farmers had poor practical knowledge of how to prevent the occurrence of *Salmonella* spp. infections, such as implementing a vaccination program, following vaccination protocols and vaccinating chickens against *Salmonella* spp. quarantining new chickens before introducing them to the flock, and conducting regular or annual evaluations of the farm’s disease status. However, 57.9% of farmers implied moderate practical knowledge regarding control of the impact of salmonellosis on production and the poultry industry (having regular health management programs with veterinarians, daily health checks for poultry, and using antibiotic medication for treatment, growth promotion, prophylaxis, and therapy purposes) (Table 2). Interestingly, 100% of poultry farmers used antibiotics for the treatment of any infectious diseases in poultry production.

Concerning mycotoxins, the majority of farmers (63.2%) had poor basic knowledge of poultry feed management, while only 7.9% demonstrated good understanding, and 28.9% had a moderate level of awareness. Key aspects of feed management include recognizing issues like moisture during storage, the presence of humidity in feed, improper handling and storage of feed, and the potential for fungal growth on poultry feed. Farmers also had poor basic knowledge of the risks of the proximity of contamination areas with mold to increasing fungal growth and the use of additives like propionic acid and calcium propionate to prevent fungal growth and improve feed quality.

Additionally, 65.7% of farmers had limited basic knowledge about the factors that contribute to mycotoxin contamination. Many were unaware that damage to grains, such as sorghum, before or during harvest increases their susceptibility to mycotoxins. They also lacked the understanding that fungi are a source of mycotoxins, poor hygiene during production can result in contamination, moisture exposure can lead to mycotoxin development in feed, and the presence of rodents or insects can promote fungal growth on feed (Table 2).

This study revealed that 73.7% of farmers had inadequate basic knowledge about identifying signs of mycotoxin-contaminated feed (abnormal discoloration, consistency, and bad odor, mold growth, and clumping of feed as indicators of fungal growth and mycotoxin contamination). Moreover, 60.5% had poor basic knowledge about the health impacts of mycotoxins on poultry and humans (the susceptibility of chickens to mycotoxins, the poultry health effects of fungal growth, and the potential of mycotoxin contamination to cause illness and poor growth in chickens). Additionally, they were unaware that mycotoxin-exposed chickens produce fewer eggs and that aflatoxins from contaminated poultry meat and eggs can be transmitted to humans (Table 2).

Regarding practical knowledge, the survey results indicated that 65.8% of farmers had moderate practical knowledge of the proper way to store poultry feed, such as using proper storage places, closing feed containers completely, avoiding shared equipment between batches and stored contaminated feed, and storing feed under dry and clean conditions for prolonged periods. Moreover, 68.4% had moderate practical knowledge concerning strategies of preventing mycotoxin occurrence, such as frequently checking for mold growth and spoilage, using drying and sorting strategies, keeping feed cool, and disposing of contaminated feed. However, 52.6% of farmers had poor practical knowledge about actions to prevent contaminated feed/food, including buying from reputable sources, ensuring hygienic production, and discarding contaminated feed/food. Moreover, 55.3% had poor practical knowledge of how to control mycotoxin impacts, for instance, by examining products for contamination, seeking medical attention for aflatoxin exposure, reporting exposure to healthcare providers, and properly disposing of contaminated meat and eggs (Table 2).

### 3.3. Basic and Practical Knowledge Scores About Salmonellosis and Mycotoxins

The overall score results indicated that 60.5% of the participants had poor basic knowledge (scored below the mean), while 39.5% exhibited good basic knowledge (scored above the mean) on salmonellosis. Regarding practical knowledge about preventing and controlling *Salmonella* spp. infections on poultry farms, 52.6% of the respondents had poor practical knowledge (scored below the mean), and 47.4% scored above the mean.

Concerning mycotoxins, the scores results showed that 52.6% and 47.4% of the farmers had below-average and above-average basic knowledge scores, respectively. Concerning practical knowledge, 55.3% of the poultry farmers showed acceptable practical knowledge (scored above the mean), while 44.7% of the respondents had applied unacceptable practical knowledge (scored below the mean) (Table 3).

### 3.4. Biosecurity Scores

Based on the percentage of scores below and above world standards (WS), the biosecurity scoring parameters revealed that a significant proportion of scores fell below WS, with 52.6% of external biosecurity scores, 89.5% of internal biosecurity scores, and 65.8% of total biosecurity scores that failed according to global benchmarks. The overall mean biosecurity score of poultry farms in Jimma, Ethiopia, was low at 41.7. The external biosecurity scores were below world biosecurity scores, with an overall mean score of 44.9. Moreover, all internal biosecurity parameters scored below the global standard. On the scoring platform, the backyard had a relatively higher external biosecurity score of 71.4% compared to broilers and layers. However, all three scoring platforms showed that internal biosecurity was the weakest aspect, with scores falling below global standards. Moreover, the broiler scoring platform had overall scores rated below international biosecurity benchmarks in all scoring parameters (Table 4).

### 3.5. Correlation Matrix Between Basic and Practical Knowledge of Salmonella spp. Infection and Mycotoxins and Overall Biosecurity

The Spearman correlation (rₛ) analysis showed weak correlations between biosecurity practices and the basic and practical knowledge scores of *Salmonella* spp. infections. Moreover, the correlations between overall biosecurity scores and the control of mycotoxins among poultry farmers were weak. The results indicate that the relationships between basic and practical knowledge regarding *Salmonella* prevention, mycotoxins, and overall biosecurity practices are generally weak and inconsistent. Furthermore, none of the correlations were statistically significant, as all *p*-values were greater than 0.05 (Table 5).

## 4. Discussion

The study highlights a significant basic knowledge gap among poultry farmers on *Salmonella* spp. infections and its impact on poultry health and farm operations, specifically a lack of understanding regarding the impact of salmonellosis on chickens. Moreover, 78.9% of respondents had a poor understanding of how salmonellosis affects the production and economy of poultry farms. These findings align with results from other developing African countries, such as Nigeria, where most respondents had limited awareness of *Salmonella* infections and their effects on poultry [49]. In contrast, certain developed European countries, such as Ireland, reported higher awareness among poultry farmers: 94% of participants understood that *Salmonella* could cause illness in both poultry and humans, recognized its potential presence on all parts of the egg, and knew that salmonellosis is not exclusively linked to poultry [50].

In contrast to this study by Conway et al. [47], it was reported that a significant majority of farmers were unaware of the zoonotic nature of *Salmonella* spp. This discrepancy suggests that while some farmers have a general awareness of *Salmonella*’s impact, there remains a substantial gap in understanding its zoonotic potential and modes of transmission. This inconsistency both between studies and within the study underscores the critical need for targeted educational initiatives. It suggests that while basic awareness of *Salmonella* is present among many farmers, there is still a lack of depth in their understanding, particularly regarding its zoonotic risks and transmission. Addressing these gaps through education and training is essential to improving poultry health management and reducing the risks posed by *Salmonella* spp.

The results showed that 50.0% of poultry farmers had lower practical knowledge in preventing and 57.9% had moderate practical knowledge in controlling *Salmonella* infections, with significant shortcomings in farm management, hygiene, and vaccination programs. While many farmers had health management programs to mitigate economic impacts, these practices did not extend to effective prevention. Notably, all farmers reported using antibiotics as a control approach, a result that is alarming as uses are related to antimicrobial resistance development [51]. This also aligns with Kauber et al. [52], who found that backyard flock owners often failed to consistently reduce the risk of *Salmonella* and other zoonotic diseases in the USA. Despite good knowledge about *Salmonella* spp. infections, farmers relied heavily on antibiotics, indicating a gap between awareness and action. This highlights the need for targeted interventions to ensure the practical application of knowledge in farm management. Similarly to salmonellosis, the results highlight significant gaps in basic knowledge among poultry farmers regarding the risks of mycotoxins, with 63.2% demonstrating low scores on awareness. Specifically, farmers showed limited understanding of poultry feed management, lacked awareness of key factors leading to mycotoxins contamination and were unable to recognize their clinical signs. Moreover, the fact that 60.5% of farmers were not aware of the health risks mycotoxins pose to both poultry and humans further emphasizes the need for enhanced education and awareness. This situation is not unique, as similar findings have been reported across various African countries. There was extremely low awareness of aflatoxins among poultry farmers and feed processors in Nigeria and Rwanda [38,53,54]. Moreover, a higher percentage of poultry and urban farmers were unaware of aflatoxins and their dangers in Ethiopia and Kenya [55,56]. Additionally, over half of smallholders in Ghana, Malawi, and Tanzania lacked knowledge on mitigating aflatoxins during pre- and post-harvest stages, and perceived aflatoxin contamination as uncontrollable and of low severity [57,58,59,60]. This widespread lack of awareness is likely contributing to the neglection of feed quality by some poultry farmers and feed processors. Therefore, there is a critical need to enhance awareness and education on mycotoxins among poultry farmers to improve their basic and practical knowledge in preventing and controlling the impact of these toxins.

Notwithstanding the poor basic knowledge, the survey results indicated that poultry farmers had moderate practical knowledge in storing poultry feed (65.8%) and preventing the occurrence of mycotoxins (68.4%). However, there are still significant areas of concern, as over half exhibit poor practical knowledge in handling contaminated feed and food and controlling the impacts of mycotoxins on both humans and chickens. These results are consistent with previous reports indicating that poor handling and storage practices significantly increase the risk of mycotoxin contamination, as many farmers neglect mycotoxin control due to limited awareness of health risks, leading to suboptimal practices and hindering effective countermeasures [38,61,62].

The biosecurity scoring parameters revealed that a significant proportion of scores fell below global standards, with 52.6% of external biosecurity scores, 89.5% of internal biosecurity scores, and 65.8% of total biosecurity scores failing to meet benchmarks. Additionally, Ameji et al. [63] identified poor biosecurity practices such as the absence of footbaths or hand wash stations, disposing of poultry litter in refuse dumps, using poultry litter as manure, and sourcing rearing stock from live bird markets and other unknown sources in Nigeria. 

The overall mean biosecurity score of poultry farms in Jimma town, Ethiopia, was notably low at 41.7. This score is a bit lower than the overall biosecurity score of 43.1 reported for central Ethiopia [9] and significantly below the average global score was 64, with 63 and 64 for external and internal biosecurity, respectively [62]. Specifically, the overall mean score for external biosecurity and all internal biosecurity parameters scored below the global standard. Similar trends were noted by Waktole et al. [9], who also found that internal biosecurity scores in Ethiopia were below global averages. Tsegaye et al. [40] highlighted that most small and medium commercial farms in Ethiopia operate under low biosecurity levels. This pattern is consistent with findings from other regions, where poor biosecurity practices have been reported in Nigeria, Egypt and Cameroon [64,65,66]. Moreover, the current biosecurity levels in Jimma are substantially lower than those reported in developed European countries [67]. The study highlights an urgent need to improve biosecurity practices on poultry farms in Ethiopia, contrasting sharply with the strict regulations in place in developed countries’ poultry industries. 

The backyard scoring platform had a relatively high external biosecurity score of 71.4%. Align to this, Gomez and Mbaga [68] found that Tanzanian farmers moderately applied biosecurity measures to prevent disease outbreaks. In Nigeria, farmers demonstrated good knowledge of basic biosecurity practices, particularly in sanitation [69]. However, despite a generally detailed understanding of disease risk factors, biosecurity measures were often lacking or ineffective [5]. 

The results indicated all three scoring platforms showed that internal biosecurity was the weakest aspect, with scores falling below global standards. Moreover, the broiler scoring platform had overall rated below international biosecurity benchmarks in all biosecurity scoring parameters. In agreement with this 80% of small-scale broiler farms lacked adequate biosecurity measures in Bangladesh [70] and were not widely adopted across all classes of smallholder poultry farmers in Kenya [71]. This study identified a generally very low level of biosecurity that needs improvements on visited poultry farms, in contrast to developed countries where strict national regulations on the poultry industry are practiced. The correlation analysis showed weak correlations between biosecurity practices and the basic knowledge and practical knowledge scores related to mycotoxins and the control of *Salmonella* infections among poultry farmers. The results indicate that the relationships between basic knowledge and practical knowledge regarding *Salmonella* prevention, mycotoxins and biosecurity practices are generally weak and inconsistent. Furthermore, none of the correlations were statistically significant. However, improving basic knowledge, adopting managemental strategies, developing good practical knowledge around mycotoxin control and strengthening both external and internal biosecurity measures are key to reducing the incidence of *Salmonella* spp. infections in poultry farms by enhancing management practices [72]. This finding also highlights the close relationship between farm biosecurity measures and *Salmonella* spp. infections in poultry farms. Infectious diseases are primary issues due to a lack of implementation of proper management and strict biosecurity measures. To prevent the occurrence of salmonellosis, farms should adopt management strategies and preventive programs.

This also makes the farmers vulnerable to disease as they are not adhering to all the necessary biosecurity measures [73,74]. Focusing on education about preventive measures on feed quality, vaccination, and biosecurity could significantly reduce the prevalence of *Salmonella* spp. infectious and zoonotic diseases in poultry farming. 

## 5. Conclusions

This study provides crucial baseline data on the knowledge and practices of poultry farmers in Jimma, Ethiopia, regarding poultry salmonellosis, mycotoxins, and biosecurity scores. The findings reveal that farmers in the study areas have poor basic knowledge and practical knowledge scores related to salmonellosis and mycotoxins. The first biosecurity assessment using Biocheck.UGent recorded external, internal, and overall biosecurity scores clearly below global standards. The correlation results suggest that inadequate basic knowledge and practical knowledge regarding mycotoxins and inadequate biosecurity contribute to the occurrence of poultry salmonellosis. The significant gaps in awareness and biosecurity underscore the need for implementing robust biosecurity measures on all farms and providing comprehensive training for farm owners and employees since effective management and strict protocols are key to reducing disease risk, emphasizing the importance of best practices in hygiene, animal handling, and feed storage. Additionally, more educational and awareness programs are essential to improve husbandry, hygiene practices, and disease prevention among poultry farmers.

## Figures and Tables

**Table 1:** Basic and practical knowledge scores of poultry farmers on salmonellosis in Jimma, Ethiopia (*n* = 38).

BASIC KNOWLEDGE VARIABLES	Mean Score ± SD	Category	Frequency	%
**Farmer’s awareness of salmonellosis**	1.55 ± 0.55	low	26	68.4
		medium	12	31.6
		high	0	0.0
**Impact of salmonellosis on production and poultry industry**	1.63 ± 0.58	low	30	78.9
		medium	8	21.1
		high	0	0.0
**PRACTICAL KNOWLEDGE VARIABLES**				
**Farm management and hygiene practices**	1.89 ± 0.72	low	26	68.4
		medium	12	31.6
		high	0	0.0
**Prevention of *Salmonella* occurrence**	1.28 ± 0.56	low	19	50.0
		medium	14	36.8
		high	5	13.2
**Control of *Salmonella* impact on farms**	1.16 ± 0.36	low	15	39.5
		medium	22	57.9
		high	1	2.6

**Table 2:** Basic and practical knowledge practices scores of poultry farmers on mycotoxin contamination in Jimma, Ethiopia (*n*= 38).

BASIC KNOWLEDGE VARIABLES	Mean Score ± SD	Category	Frequency	%
Feed management	2.10 ± 0.50	low	24	63.2
		medium	11	28.9
		high	3	7.9
Predisposing factors to mycotoxin contamination	1.73 ± 0.54	low	25	65.7
		medium	11	28.9
		high	2	5.3
Signs of mycotoxin contamination	1.44 ± 0.50	low	28	73.7
		medium	9	23.7
		high	1	2.6
Poultry and human health impact of mycotoxins	1.47 ± 0.50	low	23	60.5
		medium	13	34.2
		high	2	5.3
PRACTICAL KNOWLEDGE VARIABLES				
Storage conditions	2.50 ± 0.64	low	9	23.7
		medium	25	65.8
		high	4	10.5
Action on contaminated feed/food	2.34 ± 0.62	low	20	52.6
		medium	17	44.7
		high	1	2.6
Prevention strategy to reduce mycotoxin contamination	1.65 ± 0.48	low	10	26.3
		medium	26	68.4
		high	2	5.3
Ways to control/reduce the impact of mycotoxin contamination	1.52 ± 0.55	low	17	55.3
		medium	21	44.7
		high	0	0.0

**Table 3:** Distribution of poultry farmers’ basic and practical knowledge scores on *Salmonella* spp. infections and mycotoxins based on the mean in Jimma, Ethiopia (*n* =38).

	Basic Knowledge Score	Practical Knowledge Score
**Mycotoxins**	Mean score ± SD: 1.61 ± 1.19	Mean score ± SD: 2.61 ± 1.17
	*Number of respondents*	
Below average	20 (52.6%)	17 (44.7%)
Above average	18 (47.4%)	21 (55.3%)
***Salmonella* spp. infectious**	Mean score ± SD: 0.52 ± 0.72	Mean score ± SD: 1.57 ± 1.28
	*Number of respondents*	
Below average	23 (60.5%)	20 (52.6%)
Above average	15 (39.5%)	18 (47.4%)

**Table 4:** Biocheck.UGent™ biosecurity scoring platform and parameters and results of 38 farms, Jimma, Ethiopia.

Scoring Platform	Biosecurity Scoring Parameters	Mean ± SD	Below WS *n* (%)	Above WS *n* (%)	WS in %
**Backyard**	**EXTERNAL BIOSECURITY**	42.0 ± 13.2	6 (28.6)	15 (71.4)	31
	Purchase of eggs or one-day-old chicks	52.0 ± 19.0	13 (61.9)	7 (33.3)	66
	Purchase of laying hens	7.2 ± 12.5	4 (19.0)	17 (81.0)	29
	Depopulation and transport of poultry and products	39.4 ± 19.1	17 (81.0)	4 (19.0)	56
	Feed and water supply	43.4 ± 24.7	1 (4.8)	20 (95.2)	23
	Manure and carcass removal	30.6 ± 23.6	16 (76.2)	5 (23.8)	39
	Visitors and farm personnel	18.1 ± 16.6	10 (47.6)	11 (52.4)	14
	Infrastructure and biological vectors	51.3 ± 14.3	0 (0.0)	21 (100.0)	19
	Location of the farm	77.7 ± 18.1	0 (0.0)	21 (100.0)	33
	**INTERNAL BIOSECURITY**	21.9 ± 12.2	17 (81.0)	4 (19.0)	33
	Disease management	26.2 ± 16.6	15 (75.4)	6 (28.6)	31
	Cleaning and disinfection	18.8 ± 10.5	19 (90.5)	2 (9.5)	35
	**TOTAL BIOSECURITY SCORES**	38.1 ± 12.5	9 (42.9)	12 (57.1)	32
**Layers**	**EXTERNAL BIOSECURITY**	2.0 ± 12.3	10 (76.9)	3 (23.1)	58
	Purchase of eggs or one-day-old chicks	2.0 ± 00.0	14 (36.8)	7 (18.4)	54
	Purchase of laying hens	35.2 ± 29.7	7(53.8)	2(15.4)	73
	Depopulations and transport of hens	19.3 ± 21.6	12 (92.3)	1 (7.7)	57
	Transport of eggs	31.2 ± 4.7	13 (100.0)	0 (0.0)	42
	Feed and water	38.1 ± 20.6	7 (53.8)	6 (46.2)	50
	Manure and carcass removal	33.3 ± 7.8	13 (100.0)	0 (0.0)	48
	Visitors and farm workers	67.6 ± 20.4	4 (30.8)	9 (69.2)	63
	Material supply	90.8 ± 17.5	3 (23.1)	10 (76.9)	72
	Infrastructure and biological vectors	65.6 ± 9.8	7 (53.8)	6 (46.2)	66
	Location of the farm	73.5 ± 22.3	6 (46.2)	7 (53.8)	64
	**INTERNAL BIOSECURITY**	31.6 ± 16.8	13 (100.0)	0 (0.0)	68
	Disease management	54.6 ± 21.0	10 (76.9)	3 (23.1)	72
	Cleaning and disinfection	26.7 ± 12.7	13 (100.0)	0 (0.0)	68
	Materials and measures between compartments	33.8 ± 29.2	3 (23.1)	4 (30.8)	64
	Egg management	37.5 ± 11.9	13 (100.0)	0 (0.0)	62
	**TOTAL BIOSECURITY SCORE**	41.7 ± 12.5	12 (92.3)	1 (7.7)	63
**Broilers**	**EXTERNAL BIOSECURITY**	50.0 ± 8.0	4 (100.0)	0 (0.0)	65
	Purchase of broilers	61.0 ± 22.7	2 (50.0)	2 (50.0)	63
	Depopulation of broilers	40.0 ± 10.9	4 (100.0)	0 (0.0)	57
	Feed and water	13.0 ± 7.6	4 (100.0)	0 (0.0)	57
	Manure and carcass removal	6.3 ± 6.3	4 (10.0)	0 (0.0)	55
	Visitors and farm personnel	60.5 ± 16.1	2 (50.0)	2 (50.0)	69
	Material supply	78.0 ± 25.4	2 (50.0)	2 (50.0)	68
	Infrastructure and biological vectors	58.5 ± 14.1	4 (100.0)	0 (0.0)	77
	Location of the farms	80.8 ± 15.9	1 (75.0)	3 (75.0)	65
	**INTERNAL BIOSECURITY**	47.8 ± 7.7	4 (100.0)	0 (0.0)	70
	Disease management	59.8 ± 10.5	4 (100.0)	0 (0.0)	77
	Cleaning and disinfection	26.5 ± 7.5	4 (100.0)	0 (0.0)	65
	Materials and measures between compartments	53.5 ± 35.6	4 (100.0)	0 (0.0)	72
	**TOTAL BIOSECURITY SCORE**	47.3 ± 7.4	4 (100.0)	0 (0.0)	66
**Total**	Overall external biosecurity	44.9 ± 12.3	20 (52.6)	18 (47.4)	63
	Overall internal biosecurity	31.6 ± 16.8	34 (89.5)	4 (10.5)	64
	**OVERALL BIOSECURITY**	41.7 ± 12.5	25 (65.8)	13 (34.2)	64

Key: WS—World standard/score.

**Table 5:** A correlation among mycotoxins, biosecurity, and *Salmonella* infection on poultry farmers’ awareness and practices, Jimma, Ethiopia.

Correlations Among Variables	rₛ	*p*-Value	95% CI
Overall biosecurity vs. BPK Mycotoxins	−0.25	0.12	−0.52–0.07
Overall biosecurity vs. BPK *Salmonella*	0.17	0.30	−0.16–0.46
BPK Mycotoxins vs. BPK *Salmonella*	0.06	0.69	−0.26–0.38

BPK—Basic and Practical Knowledge.

## Data Availability

The original contributions presented in this study are included in the article. Further inquiries can be directed to the corresponding author.
